# Expectations and concerns about transitioning to face-to-face learning among Korean nursing students: A mixed methods study

**DOI:** 10.1371/journal.pone.0296914

**Published:** 2024-01-18

**Authors:** Hyeongsuk Lee, Hye Jin Yoo

**Affiliations:** 1 College of Nursing, Gachon University, Incheon, Korea; 2 College of Nursing, Dankook University, Cheonan, Korea; Tsinghua University, CHINA

## Abstract

Owing to the coronavirus disease pandemic, nursing education materials were developed for online use. However, as nursing involves working with human beings, the experience of face-to-face learning is important. This study investigated the learning satisfaction and anxiety experienced by nursing students based on their learning methods, expectations, and concerns about transitioning entirely to face-to-face learning. Using a mixed-methods design, 120 and 14 third- and fourth-year nursing students in Korea completed an online survey and individual interviews, respectively. Data were collected from July to August 2022 to assess nursing students’ learning satisfaction, anxiety, expectations, and concerns based on their learning method. Learning satisfaction was 3.96±0.68 out of 5; the students who experienced “online lectures only” had significantly higher overall satisfaction (F = 3.22, p = .002), nursing lectures satisfaction (F = 2.01, p = .046), and nursing practicum satisfaction (F = 2.19, p = .031). Anxiety was measured using the Generalized Anxiety Disorder-7 tool and was evaluated at the “minimal level,” with an average score of 3.46 ± 4.80 out of 21. From the qualitative results obtained through interviews, we derived three categories and nine subcategories. These categories include: the burden of unfamiliar learning situations that are difficult to predict, considerations about face-to-face learning needed to improve learning satisfaction, and the turning point that offsets the sense of deprivation during college life. The qualitative results provided evidence for determining specific goals for face-to-face learning that reflected the opinions of nursing students. To successfully transition to face-to-face learning, it is essential to consider a combination of student efforts, professors’ attention, and university-level support to develop a learning approach that combines the strengths of both online and face-to-face learning. Maximizing the benefits of online learning, such as integrating face-to-face and online learning through repetitive reviews of recorded videos of face-to-face sessions at their own time, can effectively reduce students’ burdens and anxiety and increase their learning satisfaction.

## Introduction

The coronavirus disease (COVID-19) pandemic has presented an opportunity to bring about a total change in the techniques used in nursing education [1, 2]. The traditional form of learning is face-to-face learning, which is defined as instructors and students interacting in-person in the same physical space, while online learning is conducted using information and communication technology so that instructors and students can be in different locations and not need to meet in-person [3]. In the field of nursing, online learning was only gradually being implemented prior to the COVID-19 pandemic because it limited students access to healthcare institutions, there has been an increase in patient rights, and the Fourth Industrial Revolution has caused rapid technological developments. However, the scope of online learning rapidly increased with the onset of the COVID-19 pandemic [2, 4]. As part of that increase, the number of clinical practicums have been limited, or practicums have occurred in a non-hospital environment, such as on campus or online [5–7].

From March 2020 to June 2022, in accordance with the national-level social distancing reinforcement policy imposed in response to the COVID-19 pandemic, South Korea implemented either online learning or a combination of both face-to-face and online learning in all its universities [8]. Universities delivered online learning in various ways, such as through real-time video lectures using Zoom or WebEx, and recorded lectures using existing video media [9].

Before the COVID-19 pandemic, online lectures in the United States and Europe were experiencing growth and development; however, online learning at Korean universities constituted only approximately 1% of the total university learning [10, 11]. Due to the concerns about potential infection with the rapidly spreading coronavirus disease, online learning was introduced during the pandemic without sufficient preparation, which created challenges in learning for both professors and students [5, 9].

Initially, it was expected that online learning would only be implemented on a temporary basis. However, prologued usage has caused universities to make intensive efforts to establish a stable online education environment [4]. Between 2020 and 2022, online education in university has become a new way of learning rather than merely an alternative to face-to-face learning, and many of the limitations of online learning have been addressed through various experiences [9].

At the end of April 2022, Korea began to recover from the impact of the COVID-19 pandemic, and its social distancing restrictions were completely lifted [12]. Trial and error had allowed the online learning process, once unfamiliar, to become an established and stable learning environment; however, students are now faced with the complex situation of reverting to face-to-face learning.

With the confusing changes in the educational environment and methods presented above, there is a lack of understanding of nursing students’ expectations and concerns about returning to face-to-face learning. Examining how prepared students are to transition to face-to-face learning when they have never experienced it before is necessary to effectively achieve the purpose of nursing education, i.e., to nurture prepared clinical nurses. Such insight could provide guidance on what nursing college professors should consider and how they should prepare to transition to face-to-face learning.

Currently, as studies on nursing education related to COVID-19 focus on practical education, such as online practicum experience [6] and clinical practicum experience [13], there is a limit opportunity for a balanced perspective between lectures and practicum of nursing students. Previous studies [14] on nursing students’ anxiety and coping strategies do not fully reflect the Korean situation and its specific educational systems, COVID-19 response policies, and cultural contexts. Therefore, this study aims to understand the learning satisfaction and anxiety of nursing students about transitioning exclusively to face-to-face learning after the pandemic through their learning methods, expectations, and concerns. Specifically, this study attempts to answer the following research question: “What are nursing students’ expectations and concerns regarding the transition to face-to-face learning, and what characteristics of students are associated with their anxieties and satisfactions during the change in learning methods?

## Methods

### Study design

This study employed a mixed-methods design that combines quantitative investigation to examine the learning satisfaction and anxiety of nursing students and qualitative individual interviews to gain a deeper insight into the research question regarding the transition to face-to-face learning [15].

### Setting and participants

The quantitative study participants were 109 nursing students; the sample size was determined by calculating a significance level of .05, a power of .90, and an effect size of 0.3 (medium size), using G*power 3.1.9 version, based on a previous study [16]. Originally, 120 participants were considered, and a 10% dropout rate was anticipated [17]. The qualitative study participants comprise 14 students from the quantitative survey participants who voluntarily participated in the interview.

Inclusion criteria were as follow: (1) third- and fourth-year nursing students; (2) no learning experience that consisted entirely of face-to-face learning during the semester; and (3) voluntarily participated in the study. Exclusion criteria were nursing students whose entire course load was currently face-to-face learning.

### Measurements

#### Participants’ characteristics

General characteristics included age, gender, academic year, residence type, and commute time; the previous semester’s learning methods included online learning methods and a portion of face-to-face learning. Additionally, the preferred learning method in the next semester and plans to change residence type were surveyed.

In this study, nursing education included the following.

- Face-to-face learning: face-to-face lectures and clinical practicums in the clinical field, such as healthcare institutions.
- Online learning: online lectures, including pre-recorded or real-time non-face-to-face lectures, and online practicums, such as simulation programs and nursing skills videos.
- Nursing lectures: face-to-face and online lectures.
- Nursing practicum: clinical and online practicums.

#### Learning satisfaction

To measure overall learning satisfaction and satisfaction related to nursing lectures and practicum, an 8-item tool developed by Kim and Pak [18] was used. Items are rated on a 5-point Likert scale, and scores range from 1 (strongly disagree) to 5 (strongly agree), with higher scores representing higher learning satisfaction. Kim and Pak [18] conducted an exploratory factor analysis using principal component analysis and confirmed the sufficient validity of the learning satisfaction measurement. During development, Cronbach’s α was .96; in this study, Cronbach’s α was .91.

#### Anxiety

The Generalized Anxiety Disorder 7-item (GAD-7) is a self-report tool with high reliability and validity developed to identify generalized anxiety. Löwe et al. [19] conducted a confirmatory factor analysis to support the validity of GAD-7 and confirmed its applicability for measuring anxiety in the general population. During development, its Cronbach’s α was .89; in this study, it was .95. Its seven items are scored from 0 to 3 points, and the score range is 0–21 [17]. The higher the total score, the higher the degree of anxiety. Total scores of 0–4, 5–9, 10–14, and 15–21 indicate “minimal,” “mild,” “moderate,” and “severe” anxiety, respectively. As the anxiety scale is highly skewed, Tabachnick and Fidell [20] recommend using a log transformation for substantial skewness. Hence, a log transformation (after adding a constant) was performed [21].

### Data collection

Data were collected in South Korea from July 17 to August 20, 2022. For quantitative data, recruitment documents were posted on the online bulletin board, and an online survey was conducted through an online Google questionnaire. The questionnaire was measured by self-report and took approximately 5–10 minutes.

Qualitative data were obtained through semi-structured individual interviews with the 14 individuals who voluntarily participated. The interview questions were based on previous studies [6, 22], and were reviewed by three nursing college professors with more than 10 years of experience as well as three third- and fourth-year nursing students. The interview questions consisted of the impact of non-face-to-face learning on life, expectations and concerns about face-to-face learning, support needed for face-to-face learning, and the effects of face-to-face classes in nursing. The interviews took 60–90 minutes per student and were conducted in a quiet seminar room where the participants could not be overheard so they would feel comfortable speaking. There were no dropouts, and data saturation occurred with the 14 participants. All interviews were audio recorded with consent and field notes were taken.

### Data analysis

The quantitative data were analyzed using IBM SPSS Statistics version 25.0 (IBM Corporation, Armonk, NY, USA). As per the research objectives, the characteristics of students that were associated with the anxiety related to the change in learning methods as well as learning satisfaction with the previous semester’s learning method were investigated. Differences in learning satisfaction and anxiety were analyzed according to general characteristics using independent t-tests and one-way ANOVA. The Pearson correlation coefficient was used for the relationships between variables, and the Scheffe test was used for post hoc testing.

The qualitative data were analyzed using inductive content analysis [23]. Throughout the preparation and organization stages, the analysis unit was selected, meaningful words or phrases were coded, the extracted codes were classified according to characteristics, and the names were nine subcategorized. Afterwards, similar subcategories were grouped together to establish three categories. In the reporting stage, the contents of subcategories and categories were described by comparing them with the original data.

Qualitative findings are useful for interpreting results that are difficult to fully reveal through quantitative analysis. This study analyzed qualitative data in a way that explains and complements the quantitative results. The reliability of the analysis was confirmed through saturation of data. To achieve consistency, the analysis was conducted by two separate researchers who then reached consensus on any discrepancies through regular meetings. During the analysis, member checking was conducted to ensure content validity. To ensure neutrality, researchers maintained impartiality through efforts to avoid bias during data collection and analysis.

### Ethical considerations

This study was approved by the Institutional Review Board of Dankook University (no. DKU 2022-06-030). Participants were instructed to click on the consent icon to indicate their agreement to participate in the study before starting the survey. Prior to interviews, written informed consent was obtained from participants in the qualitative study. All personal information was protected by encryption.

## Results

### Quantitative results

#### Participant characteristics and their learning methods during the previous semester

Of the participants, 80.8% were women and 58.3% were third-year students. The mean age was 21.85 (SD 2.08) years, 59.2% lived alone, their mean commute time was 91.10 (SD 88.11) minutes, and 16.7% had plans to change their residence type before the face-to-face lectures began. Preferred online learning next semester was 54.2% (Table 1).

**Table 1:** Characteristics of the participants.

Characteristics	Categories	Mean ± SD or n (%)
Age (years)		21.85 ± 2.08
		Range 20–38
Gender	Women	97 (80.8)
	Men	23 (19.2)
Grade	Third year	70 (58.3)
	Fourth year	50 (41.7)
Residence type	Alone	71 (59.2)
	Alone during clinical practicum	22 (18.3)
	With family	18 (15.0)
	Dormitory	9 (7.5)
Commute time (minutes)^a^		91.10 ± 88.11
		Range 10–300
		(Median 40)
Plan to change residence type	No	90 (75.0)
ahead of face-to-face learning	Yes, if face-to-face	20 (16.7)
	Yes	10 (8.3)
Learning methods of previous semester		
Nursing lectures	Online lectures only	58 (48.3)
	Combination of face-to-face and online lectures	62 (51.7)
Portion of face-to-face lectures	None	58 (48.3)
	< 50%	18 (15.0)
	≥ 50%	44 (36.7)
Real-time video lectures	Yes	118 (98.3)
	No	2 (1.7)
Pre-recorded lectures	Yes	116 (96.7)
	No	4 (3.3)
Nursing practicum	Online practicum	5 (4.2)
	Combination of clinical and online practicum	105 (87.5)
	Clinical practicum	10 (8.3)
Preferred learning method	Face-to-face	31 (25.8)
in the next semester	Online	65 (54.2)
	Haven’t decided	24 (20.0)

SD, standard deviation.

^a^Average round-trip commute time from university and home (minutes).

Regarding nursing lectures, 48.3% of students only participated in online lectures, whereas in the nursing practicum, 87.5% of students experienced a combination of clinical and online practicums. Regarding face-to-face lectures, 48.3% of students had not experienced these, whereas for 36.7% of the students, more than 50% of the overall lectures had been conducted face-to-face. In terms of the methods of online lectures, the percentages of real-time video lectures and pre-recorded lectures were 98.3% and 96.7%, respectively. Concerning the preferred learning method for the next semester, 54.2% of respondents wanted online learning, and 25.8% wanted to switch entirely to face-to-face learning (Table 1).

*Anxiety about preparing for face-to-face learning*. The average anxiety score via GAD-7 was 3.46±4.80 out of 21, which indicates that 73.3% of students had a minimal anxiety level (Table 2). Commute time and anxiety showed a positive correlation (r = .20, p = .028), and students who preferred online learning had higher anxiety levels than those who preferred face-to-face learning (F = 20.47, p < .001).

**Table 2:** Anxiety according to the characteristics of participants.

Characteristics	Categories	GAD-7^a^	
		Mean ± SD	r or t or F (*p*)
Average anxiety via GAD-7		3.46 ± 4.80	
		Range 0–21	
	Minimal level (0–4)	88 (73.3)	
	Mild level (5–9)	20 (16.7)	
	Moderate level (10–14)	4 (3.3)	
	Severe level (15–21)	8 (6.7)	
Age (years)			-0.16 (.085)
Gender	Women	3.80 ± 5.17	-1.60 (.118)
	Men	2.00 ± 2.37	
Grade	Third year	3.43 ± 5.02	-0.18 (.855)
	Fourth year	3.50 ± 4.54	
Residence type	Alone	3.83 ± 5.62	1.35 (.262)
	Alone during clinical practicum	1.00 ± 1.12	
	With family	3.95 ± 3.75	
	Dormitory	2.61 ± 2.99	
Commute time (minutes)^b^			0.20 (.028)
Plan to change residence type	No	3.16 ± 4.96	2.49 (.087)
ahead of face-to-face learning	Yes, if face-to-face	4.85 ± 4.38	
	Yes	3.40 ± 4.09	
Learning methods of previous semester			
Nursing lectures	Online lectures only	3.83 ± 5.44	0.18 (.859)
	Combination of face-to-face and online lectures	3.11 ± 4.14	
Nursing practicum	Online practicum	1.00 ± 1.41	0.88 (.416)
	Combination of clinical and online practicum	3.40 ± 4.51	
	Clinical practicum	5.30 ± 7.88	
Preferred learning method	Face-to-face^c^	0.77 ± 1.33	20.47 (< .001)
in the next semester	Online^d^	5.40 ± 5.69	c<d^†^
	Haven’t decided	1.67 ± 1.86	

GAD, generalized anxiety disorder; SD, standard deviation.

^a^Mean and standard deviation are raw scores, and statistical analysis was performed after log transformation.

^b^Average round-trip commute time from university and home (minutes).

^†^Scheffe test.

#### Learning satisfaction according to the previous semester’s learning methods

The learning satisfaction was 3.94±0.77 and 3.93±0.83 in the nursing lectures and practicum, respectively (Table 3). The students who responded to “Plan to change residence type ahead of face-to-face learning” by answering “Yes, if face-to-face” showed higher overall and nursing lectures satisfaction (F = 3.47, p = .035; F = 3.35, p = .039; respectively) than those who answered “Yes.” The students who experienced “online lectures only” had significantly higher overall satisfaction (F = 3.22, p = .002), nursing lectures satisfaction (F = 2.01, p = .046), and nursing practicum satisfaction (F = 2.19, p = .031). The students who experienced the clinical practicum demonstrated more nursing practicum satisfaction than those who only took the online practicum (F = 5.54, p = .005). Students who preferred online learning in the next semester had higher overall and nursing practicum satisfaction (F = 5.87, p = .004; F = 3.18, p = .045; respectively) than those who preferred face-to-face learning.

**Table 3:** Learning satisfaction according to the characteristics of participants.

Characteristics	Overall learning satisfaction		Nursing lectures satisfaction		Nursing practicum satisfaction	
	Mean ± SD	r or t or F (*p*)	Mean ± SD	r or t or F (*p*)	Mean ± SD	r or t or F (*p*)
Age (years)	3.96±0.68	-0.15 (.095)	3.94±0.77	-0.20 (.025)	3.93±0.83	-0.03 (.787)
Gender						
Women	3.99±0.69	-0.70 (.489)	3.96±0.74	-0.50 (.620)	3.98±0.80	-1.24 (.218)
Men	3.88±0.65		3.87±0.92		3.74±0.96	
Grade						
Third year	4.00±0.72	0.50 (.616)	3.99±0.81	0.74 (.461)	3.90±0.89	-0.51 (.608)
Fourth year	3.93±0.64		3.88±0.72		3.98±0.77	
Residence type						
Alone	4.02±0.68	1.96 (.128)	3.94±0.83	1.75 (.160)	3.94±0.81	1.30 (.279)
Alone during clinical practicum	4.14±0.58		4.14±0.64		4.09±0.75	
With family	3.67±0.61		3.61±0.78		3.61±0.92	
Dormitory	3.81±0.75		4.11±0.33		4.11±1.05	
Commute time (minutes)		-0.12 (.196)		-0.05 (.580)		0.04 (.689)
Plan to change residence type ahead of face-to- face learning						
No^a^	3.98±0.63	3.47 (.035)	3.96±0.76	3.35 (.039)	3.91±0.84	1.86 (.160)
Yes, if face-to-face^b^	4.15±0.76	b>c^†^	4.15±0.75	b>c^†^	4.20±0.40	
Yes^c^	3.48±0.81		3.40±0.70		3.60±0.97	
Learning methods of previous semester						
*Nursing lectures*						
Online lectures only	4.17±0.60	3.22 (.002)	4.09±0.76	2.01 (.046)	4.10±0.72	2.19 (.031)
Combination of face-to-face and online lectures	3.78±0.71		3.81±0.77		3.77±0.91	
*Nursing practicum*						
Online practicum^d^	3.68±0.93	2.34 (.101)	4.00±0.71	1.22 (.298)	2.80±0.84	5.54 (.005) d<e,f^†^
Combination of clinical and online practicum^e^	3.95±0.67		3.90±0.77		3.96±0.81	
Clinical practicum^f^	4.38±0.60		4.30±0.82		4.20±0.79	
Preferred learning method in the next semester						
Face-to-face^g^	3.69±0.12	5.87 (.004)	3.94±0.68	0.01 (.993)	3.65±1.14	3.18 (.045)
Online^h^	4.15±0.69	g<h^†^	3.94±0.88		4.09±0.68	g<h^†^
Haven’t decided^i^	3.83±0.52		3.96±0.55		3.88±0.68	

SD, standard deviation.

^†^Scheffe test.

#### The impact of online learning and preparation for face-to-face learning

**Fig 1:**
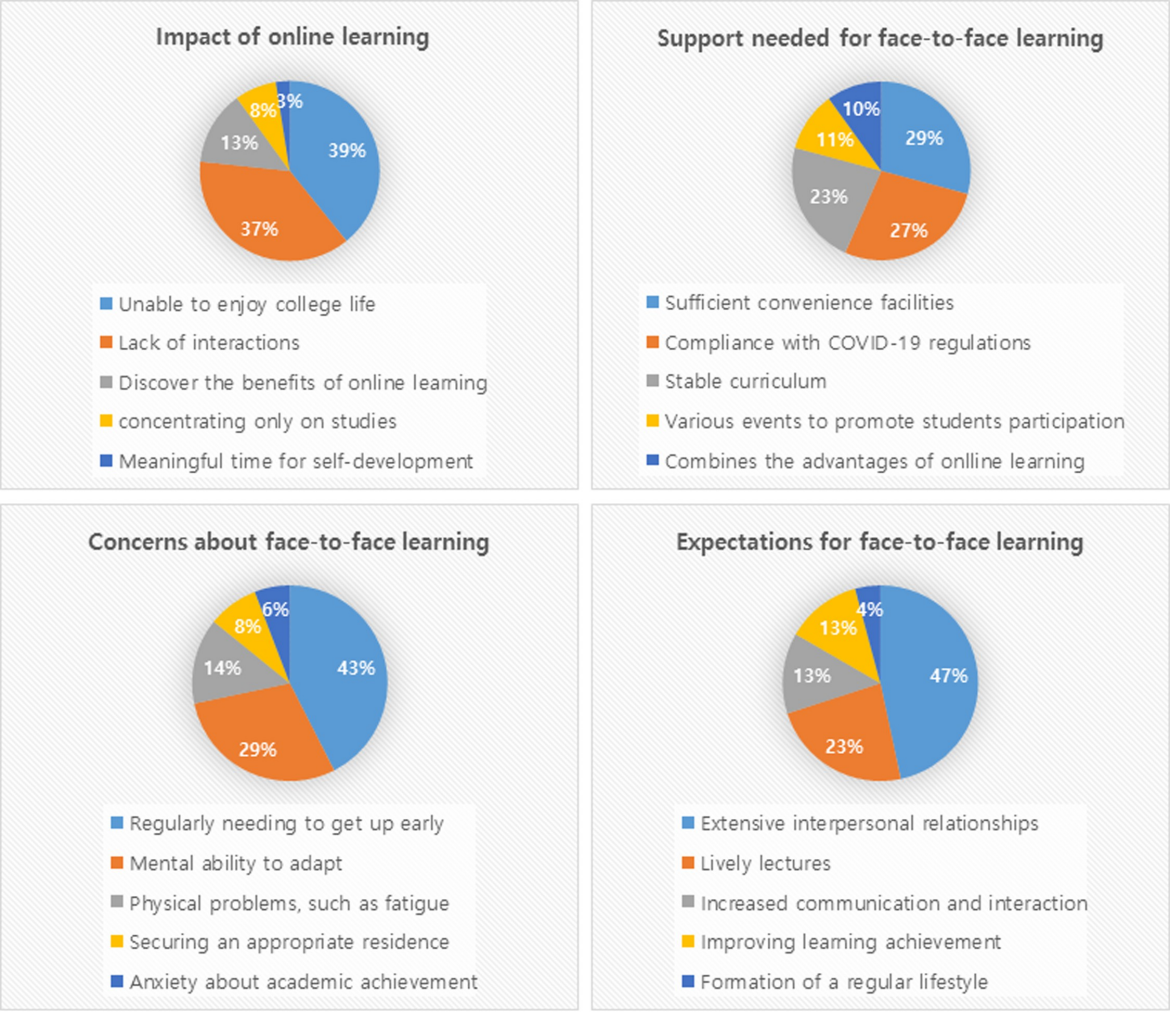
The impact of online learning and preparation for face-to-face learning.

Regarding the impact of`online learning, 39.2% of participants responded that they were “unable to enjoy college life,” and 37.9% say they experienced a “lack of interactions.” Regarding support needed for face-to-face learning, they responded with “sufficient convenience facilities” (29.2%), “compliance with COVID-19 regulations” (27.5%), and “stable curriculum” (22.5%). Participants were concerned about “regularly needing to get up early” (42.5%), “mental ability to adapt” (29.2%) ahead of face-to-face learning, but they had expectations about “extensive interpersonal relationships” (46.7%) and “lively lectures” (23.3%) (Fig 1).

### Qualitative results

The average age of the qualitative study participants was 24.57 ± 3.50 years. Of the participants, 57.1% were women and third-year students respectively. The qualitative results complemented the quantitative results; interpretations of both results were linked and explained. Nursing students’ expectations from and concerns for face-to-face learning were derived from the 26 codes, nine subcategories, and three categories (Table 4).

**Table 4:** Nursing students’ expectations and concerns for face-to-face learning (N = 14).

Categories	Subcategories	Contents
The burden of unfamiliar and unpredictable learning situations	Unexpected changes that shake up a stable life	Inconvenient changes in situations in which online learning has become typical
		Physical/mental adaptation to a regular lifestyle
		Vague fear of face-to-face learning
	Reality of having to struggle with unfamiliar relationships	Circumstances where community life is necessary
		Many issues to address besides studying (e.g., clothes, appearance, study environment, etc.)
		Body and mind exhausted from energy consumed in other ways, such as commuting time and maintaining relationships
	Concerns about learning effectiveness	Learning at a set time and place
		Unable to adjust lectures to desired pace
		Worry about securing time for self-study
Considerations in face-to-face learning needed to improve learning satisfaction	Securing a detailed student-centered guidance system	Awkward campus life not much different from freshman
		Interest and support for students adapting to changing circumstances
		Sufficient operational facilities, such as opening a 24-hour library, cafeteria operations, and shuttle bus timing
	Professors’ thorough preparation for differentiation from online learning	Sharing sufficient learning materials to help prepare for learning
		Utilization of various teaching methods to deliver information and improve concentration
		Vivid explanations linked to clinical nursing situations
	Need a solution to relieve anxiety about learning achievements	Concerns about incomplete understanding
		Craving for learning methods that can complement one-off lectures (e.g., recorded videos upload, blended learning)
Turning point that offsets the sense of deprivation of college life	Normalization of unstructured college life	Curriculum where everything takes place within the college
		Controlled behavior while being conscious of the surroundings
		Motivated learning environment through direct contact with professors and classmates
	Real learning opportunities through interactions	Immediate feedback from active communication
		Lively life like a real college student
		Team learning environment that enables smooth communication face-to-face instead of text
	Learning environment that allows for a fairer evaluation	Freed from concerns about cheating in the online space (e.g., unstable computer systems, noise, restrictions on suspicious behavior, etc.)
		Expectation of learning achievements through sufficient test time
		Transparent evaluation process that can be directly monitored by each other

#### The burden of unfamiliar and unpredictable learning situations

For students who had never had a semester where they participated in their nursing classes major entirely through face-to-face learning, the transition to this mode of learning was perceived as difficult.

*Unexpected changes that shake up a stable life*. Once again, online learning underwent a chaotic period during a stable situation. In other words, students had become accustomed to online learning after going through a chaotic period (COVID-19 pandemic), when it was first initiated; it was not welcome news.

When I first started online learning, I really longed for face-to-face learning, but after more than 2 years of online learning, I’m now used to it. On the contrary, I am not happy with face-to-face learning now. The new environment itself is very burdensome. (Participant 1)

*Reality of having to struggle with unfamiliar relationships*. Students who had become accustomed to being alone in a free environment were concerned about possibility of energy depletion caused by community life and studying in a face-to-face learning environment.

I was comfortable taking lectures and studying by myself. When attending face-to-face lectures, I find it a bit inconvenient to think about what to wear every morning and pay attention to my surroundings. I’m worried because I’m quite influenced by my surroundings, and my energy is easily depleted in relationships. (Participant 6)

*Concerns about learning effectiveness*. As students became accustomed to online learning, they chose the desired time and place to attend lectures and adapted to self-directed learning.

The biggest advantage of online learning is that there is no time constraint. In the case of recorded lectures, I adjusted my study time by choosing a learning time that suited me best and a pace that suited my level. It was really good to be able to repeatedly learn the parts that I didn’t understand well. (Participant 7)

#### Considerations in face-to-face learning needed to improve learning satisfaction

Nursing students wanted attention from their professors and for the professors to be prepared to solve difficulties related to face-to-face learning. In addition, they proposed a learning method that would retain the effects of online learning as much as possible while ensuring flexibility in face-to-face learning.

*Securing a detailed student-centered guidance system*. The students wanted help and detailed guidance regarding the university system. They hoped that they would not face any challenges in the face-to-face learning method, which needed to account for the experiences of students who had not have a typical college life previously. Additionally, they wanted the interest and support of professors to help them adapt to college life.

As I hardly ever went to university and didn’t participate in any activities, I don’t know the building’s location. I hope the professors understand our position and help us. It would also be nice if we do not face problems with operational facilities, which should be well-planned, such as opening a 24-hour library, cafeteria operations, and flexible shuttle bus timings. (Participant 13)

*Professors’ thorough preparation for differentiation from online learning*. The students emphasized developing strategies to increase differentiation from online learning through explanations related to clinical situations and various teaching methods to increase concentration, and they expected visible positive effects of face-to-face learning through this differentiation.

To be honest, considering the high convenience of and satisfaction with online learning, one’s satisfaction with face-to-face learning will inevitably decrease if its advantages are not perceptible. If the professors provide the necessary learning materials for the lectures in advance and deliver interesting lectures on clinical nursing, students will better retain the lecture content and their grades will naturally increase, right? (Participant 10)

*Need a solution to relieve anxiety about learning achievements*. Students were concerned that one-time on-site lectures might lower their understanding of learning. Through face-to-face learning, they thought about ways to relieve their anxiety.

In a face-to-face situation, I can only listen once, and it is impossible to “replay” it like a recorded lecture. So, I hope the professor will frequently check to see if the students understand well. I am a little worried about lectures disappearing owing to volatility. I think it would be better to use a mixture of online and face-to-face methods without losing the advantages of online learning (Participant 3)

#### The turning point that offsets the sense of deprivation in college life

Students favored online learning, but they also hoped to break away from the irregularities imposed by online learning and engage in vibrant, in-person learning experiences with their professors and peers. In addition, they welcomed fair evaluations without doubts and concerns.

*Normalization of unstructured college life*. Students expected that they would be able to normalize their unstructured lives through direct meetings with professors and hardworking friends, and they hoped to improve their concentration and motivation to learn.

In the case of online learning, I got distracted by lying down or doing other things in the middle. I think face-to-face learning is where universities find a place. This is the beginning of normalization. I will be stimulated by listening to live lectures and watching my friends work hard. (Participant 2)

*Real learning opportunities through interactions*. Students expected a learning environment where they could form harmonious bonds with others, as against a personal environment where they interacted only with computers. They wanted to overcome the limitations in interaction through face-to-face team activities and expressed their desire for a culture of discussion and high-quality learning.

I feel like I spent two-and-a-half years isolated on a deserted island during the most glamorous period of my life. There were many days when I felt like I was a robot; I woke up in the morning, looked at the computer, and did not leave the room until I fell asleep. In the future, I want to actively communicate and learn, rather than only through LAN lines (Participant 12)

*Learning environment that allows for a fairer evaluation*. Students hoped for a fairer evaluation without doubts about cheating, which cannot be completely guaranteed in an online test environment. They also had concerns about computer problems and the burden of shorter test times unlike in a face-to-face test.

I totally agree with face-to-face tests. When taking an online test, there are many things to pay attention to, such as sounds and direction of view, for fear of being misunderstood about cheating. I am worried that the test will be interrupted because of the wireless network problem, and I feel pressurized by the shortening of the test time (Participant 5)

## Discussion

Owing to the COVID-19 pandemic, college students were inexorably faced with restrictions regarding life on campus [8]. This study’s results show that nursing students’ acceptance of online learning was relatively positive, and their expectations and concerns coexisted rather than fully welcoming the transition to face-to-face learning.

The learning satisfaction in the previous semester among students that experienced only online learning was 3.96 out of 5, which was higher than that of the previous study (i.e., 3.31) [8]. Those who took only online lectures showed higher overall satisfaction and satisfaction with nursing lectures than those who did not. Those who preferred online learning showed higher learning satisfaction than those who preferred face-to-face learning. In Korea, professors are able to deliver stable lectures without technical difficulties by establishing an online system and leveraging the high penetration rate of wireless networks, ensuring convenient learning operation [6]. Moreover, satisfaction with online learning is judged to be a result of positive perceptions of online learning created through students’ adaptation to learning.

In the early days of the transition to online learning, there were challenges in reflecting the direction of non-face-to-face education and the needs of learners in Korea’s educational environment, where classes were primarily conducted in the lecture-style [9, 10]. However, as non-face-to-face learning emerged as the primary mode of education, the online system environment for nursing education gradually stabilized [1, 8]. This environment enables students to engage in self-directed learning through repetitive learning and gives them more time to focus on learning by removing commuting time [2]. This is explained in the same context as the study results, where students who had to change their residence type in the next semester owing to transitioning entirely to face-to-face learning showed high satisfaction with online learning. However, in flipped-learning nursing education, the results of a previous study [24] on learning satisfaction showed that the face-to-face group had higher satisfaction than the non-face-to-face group, which contradicts this study. Learning satisfaction shows a positive correlation with self-directed learning readiness and professor-student interaction [24]. The participants in this study are students who have had online-oriented learning experience, making it difficult to completely compare face-to-face and non-face-to-face learning. Accordingly, efforts will be needed to confirm qualitative and quantitative learning effects, such as satisfaction and grades, with face-to-face learning in the future.

In addition to the positive response to the online learning environment, nursing students tried to make the upcoming new semester a turning point that could offset the sense of deprivation brought about by the COVID-19 pandemic through their college life. They looked forward to a safe learning environment where they could normalize their unstructured daily schedules, experience real learning through interaction, and be fairly evaluated. In the United States and Europe, various online programs are continuously expanding as an alternative to nursing education due to a shortage of nurses [3]. Although there are advantages to online learning, non-face-to-face education may lead to maladjustment to the role of a nurse after graduation because of limited opportunities to practically apply learning in clinical situations [7, 25]. From the perspective that nursing provides person-centered care, face-to-face learning is meaningful because it can enhance interpersonal competence that was previously lacking through dynamic interactions for nursing students [26–28]. In this study, students who experienced clinical practicums showed higher satisfaction in the nursing practicum than those who experienced only online practicums. Nursing students have the opportunity to apply their nursing as well as communication and assessment skills through clinical practice [29, 30]. These factors mean that nursing is a discipline that cannot rely solely on online practicums [13]. Therefore, when nursing students who have received limited face-to-face learning as nurses, such as during the COVID-19 pandemic, comparative research with nurses who have only received face-to-face learning about nursing competencies, such as clinical adaptation and communication, will be necessary to understand the long-term impact of online learning.

Owing to the COVID-19 pandemic, various nursing education materials have been developed for online use, but nursing is a discipline that targets human beings, and the experience of nursing education through face-to-face learning is valuable [5, 28, 31]. Therefore, in the transition to face-to-face learning, there are several considerations that raise students’ expectations. First, before face-to-face learning, students’ anxiety scores were relatively low, except for students with long commute times and those who preferred online learning. In addition, the qualitative results confirmed the vague anxiety caused by returning to the traditional education method, in which students could not utilize the advantages of online learning and faced the burden of an unfamiliar college environment and community life [32]. Therefore, professors should understand the position of students, carefully guide them, and use periodic counselling to help them adjust to face-to-face learning. In addition, universities should make multifaceted efforts to support students, such as providing information on the university environment (e.g., location and facilities) and supporting students who are not familiar with college or community life through the use of seniors and mentors. Second, the vivid learning that connects nursing lectures and practicums based on clinical cases, with educational content tailored to the students’ levels and professors’ passion, is a differentiated method experienced only in face-to-face learning [28]. Therefore, it is necessary for professors to think carefully about the development and use of various teaching and learning methods, such as flipped learning, problem-based learning, and digital technology, including virtual reality, augmented reality, or metaverse for knowledge transfer and concentration improvement; such methods go beyond the convenience of online learning and the advantages of self-directed learning tools. Team activities for developing and practicing leadership skills can also be considered. Third, early in the COVID-19 pandemic, for nursing students accustomed to face-to-face learning, online learning aggravated their anxiety and stress and showed individual differences in learning effects based on students’ resilience, willingness to participate in learning, and attitudes [14, 22]. However, now that the situation is reversed, efforts are needed to compensate for the shortcomings of one-time lectures. As suggested in this study, maximizing the advantages of online learning, such as uploading recorded videos of face-to-face learning, a combination of both face-to-face and online learning [33, 34] and tele-simulation [35, 36], can be a way to lower students’ burdens and anxiety and increase learning satisfaction. Additionally, as science and technology have advanced, the development of 3D spatial education materials, such as metaverse and virtual reality, are becoming more available worldwide [37, 38]. Nursing education also needs to make continuous efforts to develop learning by actively introducing the advantages of educational methods that motivate students to learn and encourage participation.

This study has some limitations. It includes the results of nursing students without an entire semester of face-to-face learning experience; hence, care should be taken in generalizing the results. This study targeted students who voluntarily chose to participate, introducing the potential for a self-selection bias among students with either positive or negative perceptions of the transition from online to face-to-face learning. Therefore, caution is warranted when extrapolating and interpreting the research findings. Additionally, as there may be differences in experiences depending on the nursing lectures and practicum curriculum, care should be taken in interpreting the research results. In the future, in face-to-face learning situations, it is necessary to present an optimal education method suitable for students by checking the effectiveness of the nationwide curriculum operation, learning satisfaction, and degree of achievement of learning goals.

## Conclusions

This study attempted to explore the future direction of nursing education by considering nursing students’ perspectives in the context of face-to-face learning after the COVID-19 pandemic as well as preparing a nursing education environment considering their point of view. Professors should attempt to understand nursing students’ anxiety and concerns, respect their learning needs, and have an open attitude. In addition, university administrators should use this as an opportunity to build a learner-centered education system through active support for improving the learning environment, such as securing space utilization and manpower, which were reduced by online learning, and establishing convenient facilities and interactive communication systems.
